# Corporate interest groups and their implications for global food governance: mapping and analysing the global corporate influence network of the transnational ultra-processed food industry

**DOI:** 10.1186/s12992-024-01020-4

**Published:** 2024-02-22

**Authors:** Scott Slater, Mark Lawrence, Benjamin Wood, Paulo Serodio, Phillip Baker

**Affiliations:** 1 School of Exercise and Nutrition Sciences, Deakin University, Burwood, VIC Australia; 2 Institute for Physical Activity and Nutrition, Deakin University, Geelong, VIC Australia; 3 Global Centre for Preventive Health and Nutrition, Institute for Health Transformation, Deakin University, Geelong, VIC Australia; 4 Institute for Social and Economic Research, University of Essex, Wivenhoe Park, UK

**Keywords:** Ultra-processed foods, Corporate power, Multistakeholder, Partnerships, Lobbying, Front groups, Trade associations, Global food governance, Food systems

## Abstract

**Background:**

A major challenge to transforming food systems to promote human health and sustainable development is the global rise in the manufacture and consumption of ultra-processed foods (UPFs). A key driver of this dietary transition is the globalization of UPF corporations, and their organized corporate political activity (CPA) intended to counter opposition and block government regulation. UPF industry CPA and the corporate interest groups who lobby on their behalf have been well described at the national level, however, at the global level, this network has not been systematically characterized. This study aims to map, analyse, and describe this network, and discuss the implications for global food policy action on UPFs, global food governance (GFG), and food systems transformation.

**Methods:**

We conducted a network analysis of the declared interest group memberships of the world’s leading UPF corporations, extracted from web sources, company reports, and relevant academic and grey literature. Data on the characteristics of these interest groups were further extracted for analysis, including year founded, level, type, and headquarter location.

**Results:**

We identified 268 interest groups affiliated with the UPF industry. The UPF manufacturers Nestlé (*n* = 171), The Coca-Cola Company (*n* = 147), Unilever (*n* = 142), PepsiCo (*n* = 138), and Danone (*n* = 113) had the greatest number of memberships, indicating strong centrality in coordinating the network. We found that this network operates at all levels, yet key actors now predominantly coordinate globally through multistakeholder channels in GFG. The most common interest group types were sustainability/corporate social responsibility/multistakeholder initiatives, followed by branding and advertising, and food manufacturing and retail. Most corporate interest groups are headquartered where they can access powerful government and GFG decision-makers, nearly one-third in Washington DC and Brussels, and the rest in capital cities of major national markets for UPFs.

**Conclusions:**

The UPF industry, and especially its leading corporations, coordinate a global network of interest groups spanning multiple levels, jurisdictions, and governance spaces. This represents a major structural feature of global food and health governance systems, which arguably poses major challenges for actions to attenuate the harms of UPFs, and to realising of healthy and sustainable food systems.

**Supplementary Information:**

The online version contains supplementary material available at 10.1186/s12992-024-01020-4.

## Background

Ultra-processed foods (UPFs) are now a major feature of the nutrition transition [1], contributing over half of all caloric intake in some high-income countries (HICs) [2, 3], and rising rapidly in the diets of populations in both upper and lower-middle income countries [4]. This development has been driven by the industrialization of food systems, globalization and technological change, trade and investment liberalization, and the expanding commercial and political practices of the UPF industry [4, 5], all of which have resulted in UPFs becoming a central part of a ‘globalised diet’ [6]. As a result of this change in human diets, UPFs have received significant scholarly attention in recent times, with growing evidence showing that they are associated with many adverse human health outcomes [7–9], environmental and ecological harms [10–12], as well as global food governance (GFG) challenges of conflicts of interest in policy making [13–15], corporate power in the global food system [5, 16, 17], and UPF industry ‘corporate political activity’ (CPA) [18, 19]. Together, these GFG challenges represent a barrier to transforming food systems [20], particularly as the UPF industry has been increasingly positioning and presenting themselves in GFG spaces as a key ‘part of the solution’ to food system challenges, including the many food system issues and harms that UPFs cause [21].

Research demonstrates that the UPF industry (i.e.: UPF manufacturers and co-dependent corporations and industries) and their associated ‘corporate interest groups’ actively lobby key food system decision makers [22], to influence policy, and the prioritizing of UPF corporate interests in GFG spaces [19, 23, 24]. CPA and lobbying by the UPF industry are an important part of the collective term, the ‘commercial determinants of health’ (CDOH), which refers to the systems, practices, and pathways through which commercial actors drive health and equity [25]. UPF industry lobbying occurs through both direct (i.e., consultant lobbying) and indirect (i.e., memberships, partnerships, and governance positions within industry associations) [26] engagement with policy makers. The creation of ‘front groups’ [27] and ‘trade associations’ [28] form a key structural component of the UPF industry’s network of corporate interests groups, driving tactics and strategies [29] to obstruct, prevent, and weaken policies and regulations on UPFs [22]. Furthermore, this network of corporate interest groups also create influence in governance spaces through the promotion of public–private partnerships (PPP’s), multistakeholder initiatives (MSI’s) and voluntary industry self-regulation - which have been shown to be ineffective at managing the harmful effects of UPFs [5, 30]– at both the national and global level.

To understand contemporary GFG in relation to the global UPF system, we define the UPF industry as “a commercial ecosystem comprising UPF and beverage manufacturers at its core, as well as other co-dependent food supply chain sectors and industries who profit from the proliferation of UPFs, and the displacement of NOVA groups 1–3 (Unprocessed or minimally processed foods, processed culinary ingredients, processed foods) in human diets”. We take this approach because globalization has, in recent decades, seen the UPF industry increasingly expand and incorporate a wider commercial ecosystem of specialised UPF ingredient suppliers, primary producers, manufacturers, retailers, financiers, digital/tech actors, research institutions, marketing agencies and lobby groups. At the same time, this diverse range of actors all facilitate the increasing contribution of UPFs to dietary patterns around the world [32]. Growing evidence suggests that as UPF markets have grown [33], it is now clear that this wider commercial ecosystem invests in CPA lobbying [34] and sophisticated and intensive marketing strategies to protect these markets through the fostering of policy, regulatory and knowledge environments conducive to their sustained profits [4]. Furthermore, evidence now also shows that the UPF industry attempts to influence food systems governance, policy processes and scientific activities [30, 35, 36] across multiple levels, countries and regions [31, 37–39], indicating transnational coordination globally to further their interests. This is an idea recently been referred to as both an ‘architecture’ built to meet the interests of UPF corporations [40], and an ‘ultra-processing regime’ [41]. In this paper, this is conceptualized as a form of global food systems governance by transnational corporate actors which is now layered onto, draws legitimacy from, and seeks to influence the multilateral food governance system of nation states, UN agencies, and civil society groups.

Despite this understanding, and the knowledge that the corporate interest groups who lobby on behalf of the UPF industry have been well described at the national level [42], at the global level, the UPF industry’s corporate network of interest groups has not yet garnered the same scholarly attention [31], or been systematically characterized. Given the UPF industry’s role in the intersecting human and planetary health crises humanity faces [43] and the urgency and need for transformative food systems change [44, 45], we contend that this network is vital to understand, considering the power that the UPF industry now has to shape and influence food systems on a global scale, international policy fora and organizations, and global food spaces of governance. Recognising this need and research gap, the aim of this paper is to map, analyse and describe the composition and characteristics of the UPF industry’s global network of interest groups. We do this by addressing several key questions. Who are the leading UPF corporations in the world, and which corporations are central to the coordination of the UPF industry’s global political activities? Who are the corporate interest groups most connected to the network, and what are their core characteristics? How has the network evolved over time? We then discuss the characteristics of UPF industry’s network of interest groups in relation to the potential implications for global food policy action on UPFs, GFG, the food systems transformation.

## Methods

We adopted a network and documentary analysis method to meet our aim and answer this study’s key questions. This method allowed us to integrate quantitative and qualitative data drawn from a variety of sources, including web searches, business and market research databases, company reports and academic and grey literature. We proceeded through three steps: (i) data collection; (ii) categorization, network mapping, and analysis; and (iii) synthesis and discussion of results.

### Data collection

Data collection was carried out between September 2022 and February 2023 and proceeded through four steps.

#### Identifying the leading UPF corporations in the world

First, we identified the world’s leading UPF manufacturers. Using Baker et al.‘s twenty eight categories of UPFs and ultra-processed beverages (UPBs) [4], hereafter ‘UPFs’, we identified and extracted data on the top 10 manufacturers in each category using 2021 sales revenue data according to Euromonitor International’s Passport database [46]. Next, for each UPF category, we assigned each UPF manufacturer a score; ten for the highest sales revenue through to one for the lowest. Finally, the scores across all the UPF categories were summed, ranked, and the top 150 UPF manufacturers were included. We took this approach rather than simply ranking manufacturers based on their total revenues made from UPF products, because our starting assumption was that there were a core group of UPF corporations that are central to UPF systems; and thus, were also central to the corporate influence network.

To provide a more complete picture of the UPF industry, we also identified key corporations involved in UPF supply chains and co-dependent industries listed in several major reports and publications. These included UPF ingredient suppliers; sugar and seed oil commodity producers; seed, chemical and fertilizer producers; milk formula manufacturers [47]; grocery retailers, and fast food corporations [47–50]. A list of the top 150 UPF manufacturers plus the 50 UPF supply chain and co-dependent industry corporations are shown in supplementary Table 1.

#### Identifying the interest groups and organisations involved in the UPF corporate influence network

To identify the UPF industry’s corporate interest groups, we sourced ‘seed data’ from the interest group memberships declared by the top 10 UPF manufacturers identified from step 1 (e.g., PepsiCo, Nestlé, The Coca Cola Company etc.) on their websites. This involved searching each UPF manufacturers website using keywords such as ‘advocacy, membership, trade association, partners, partnerships, general disclosures, or collaborators’ until the relevant page/section was identified. These keywords were inductively identified and noted for use through searching the websites menu tabs. Often the search progressed through the UPF manufacturers webpage to ‘reports’ (i.e., environmental, social and governance (ESG), corporate social responsibility (CSR), sustainability, Global Reporting Initiative (GRI), business, governance etc.) to locate the relevant data, and when this was the case, the most recent report available was sourced.

In this step, we excluded organizations/associations which: [1] operated at the sub-national level (e.g., Bay Area Council); [2] only allowed access through a password protected member only directory; [3] the webpage was not able to be translated if not in English; and [4] were clearly not associated with the food industry. For example, Unilever is also a major cosmetic and personal care product company and is also associated with associations such as the Cosmetic Toiletry and Perfumery Association and the Personal Care Products Council. Additionally, if both global and regional organizations were captured as part of the seed data (e.g., Consumer Goods Forum and Consumer Goods Forum in Latin America) we only included data on the organization at the global level.

#### Identifying the membership links with the UPF corporations identified in step 1

We searched the websites of each corporate interest group to identify the membership links with the 200 UPF corporations identified in step one. To do this, we searched using keywords such as: about us, members, corporate partners, directors, board, scientific and working groups. If this didn’t locate the membership lists, we used the websites search function and the names of the top 30 UPF manufacturers until the information was located. As an additional step, we conducted searches to capture data on UPF industry corporate interest groups from several sources, including Lobby Facts [51], U.S. Right to Know [52], the Food Politics webpage [53], and Open Secrets [54], also using the names of the top 30 UPF corporations and the keywords lobby groups, front groups, and industry or trade associations.

#### Characteristics and additional information

We searched the websites of each corporate interest group for information including its commonly used acronym (label), level (national, regional, global), headquarters (city and country) location, and year founded. Data on the year founded was collected by searching the corporate interest groups website using the keywords, ‘founded’, ‘launched’, ‘established’ or ‘created’. If this search did not provide a year, we then searched the organization’s ‘LinkedIn’ page, the Union of International Associations webpage [55], or relevant US and EU ‘transparency register’ websites [56, 57] to locate this information. At this stage, if the website and or sufficient information couldn’t be obtained after scrolling the first five pages on Google, the corporate interest group was removed from the dataset.

### Categorization, network mapping, analysis

We used World Bank country, region, and income level groupings to categorize which country and region the corporate interest group was headquartered in [58]. As we did not identify in our searches a relevant categorization tool for the UPF industry’s corporate influence network, during data collection we inductively developed a framework for categorizing types of corporate interest groups, based on the relationship and connection to the global food system. The types, corporate interests represented, and examples are provided below in Table 1. The network graph was generated using Gephi product version 0.10.1. We generated descriptive statistics using Microsoft Excel and R version 4.2.2 (Foundation for Statistical Computing). We also used Gephi’s analysis tools to calculate degree centrality for all actors; degree centrality identifies the most central actors to the network based on the number of direct connections actors have within the overall network [59]. Direct ties are crucial for enabling swift communication, spreading information, and establishing immediate influence in a network. Degree centrality commonly aligns with other centrality indicators, highlighting various aspects of a node’s significance and its potential as a critical point within the network structure [60]. We shortened the names of some of the actors in the network to help with clarity in the network figure.

**Table 1:** Corporate interest group types

Type	Corporate interests represented	Examples
General food industry	Corporations with interests in the food industry, including those with business interests in UPF systems.	Food & Consumer Products of Canada, Federation of the Dutch Food Industry, Food Federation Germany
Primary production, processing and ingredients	Corporations with interests in the production of agricultural commodities and animals used as UPF ingredients.	European Dairy Association, International Sweeteners Association, The Whole Grains Council
Food manufacturing and retail	Corporations with interests in the processing, manufacture, and retailing of UPF products and ingredients.	FoodDrinkEurope, Consumer Goods Forum, Australian Food and Grocery Council
Branding and advertising	Corporations with interests in branding, marketing, advertising and general promotion of UPF products and ingredients.	World Federation of Advertisers, Association of National Advertisers, Food Marketing Institute
General business and trade	Corporations with interests in UPF business activities, including trade, finance, and collaborations between corporations and/or nation states.	American Chamber of Commerce to the European Union, World Economic Forum, National Association of Businessmen of Colombia
Research and science communication	Corporations with interests in studying and researching UPF products, ingredients, nutrition and food systems.	ILSI Global, Academy of Nutrition and Dietetics, Tufts University Food and Nutrition Innovation Council
Lobbying, legal and public relations	Corporations with interests in efforts to influence the political economy of the UPF systems and food related policies.	European Centre of Public Affairs, Society of European Affairs Professionals, Transatlantic Policy Network
Sustainability/CSR/MSI	Corporations with shared interests (often sustainability, social, environmental) and who collaborate to address issues of common concern.	The WEF’s Food Action Alliance, Roundtable on Sustainable Palm Oil, FReSH (WBCSD and EAT)
Specialized nutrition and baby food	Corporations with interests in specialized nutrition, breast-milk substitutes and baby foods.	International Special Dietary Foods Industries, Healthcare Nutrition Council, International Special Dietary Foods Industries
Other	Other.	American Red Cross, Ocean Conservancy, United States Agency for International Development

*Notes* CSR - corporate social responsibility; MSI - multistakeholder initiatives

## Results

Our results are divided into four sections. First, we describe the world’s leading UPF corporations, listing the top 50 and the number of membership connections to the identified corporate interest groups within the UPF corporate influence network. Second, we map and describe the global influence network in terms of the UPF corporations and corporate interest groups most connected and central to the UPF industry’s corporate influence network. Third, we show by analysing corporate interest group types and years founded how the influence network has expanded and evolved over time. Finally, we show the geographical reach and clustering of the network in terms of headquarter locations.

### The leading UPF corporations and corporate interest groups in the world

First, we identify the world’s leading UPF corporations and their declared interest group memberships. The top-10 largest UPF manufacturers by sales revenue across all UPF categories were PepsiCo Inc, followed by Nestlé, The Coca-Cola Co, Kraft Heinz Co, Unilever Group, General Mills Inc, Suntory Holdings Ltd, Danone Groupe, Mondelez International Inc, and Kellogg Co. Out of the top 10 UPF corporations, six are corporations based in the USA, three are based in Western Europe, and one is based in Japan.

**Fig. 1:**
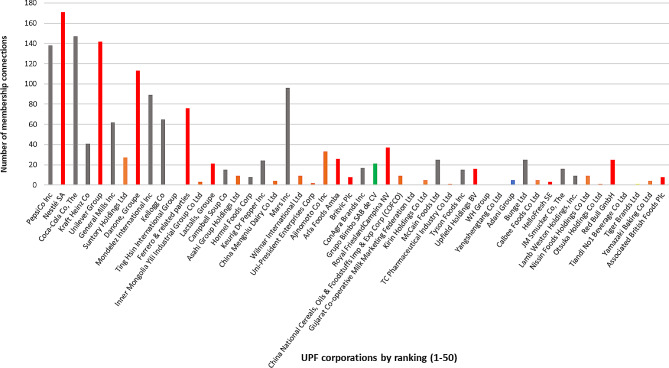
Distribution of total connections of the top 50 UPF corporations within the mapped network *Notes* Euromonitor International’s Passport database. The UPF corporations shown (top 50) are according to summed and ranked UPF company sales positions in each UPF category. The UPF and UPB categories include: Baked goods; Breakfast cereals; Confectionery; Dairy products & alternatives; Frozen processed potatoes; Ice cream & frozen desserts; Instant Pasta/Noodles; Processed Meat, Seafood and Alternatives to Meat; Ready meals; Sauces, dressings & condiments; Savoury snacks; Sweet biscuits, snack bars & fruit snacks; Sweet Spreads; Butter and Spreads; Ready Soups; Carbonated soft drinks; Concentrates; Drinking Milk Products; Functional Bottled Water; Flavoured Bottled Water; Juice drinks; Nectars; Ready to drink Coffee; Ready to drink Tea; Asian speciality drinks; Sports drinks; Energy drinks; Flavoured Powder Drinks. Colour codes shown are according to World Bank country and region groupings. Orange - East Asia & Pacific; Red - Europe & Central Asia; Green - Latin America & Caribbean; Grey– North America; Blue– South Asia

Data extracted from the 50 largest UPF manufacturer websites and the additional searches, identified 289 corporate interest groups. Of these, 21 did not allow access to the membership list or directory without login details and/or passwords and were excluded, leaving 268 corporate interest groups for analysis. From these 268 corporate interest groups, 3366 connections with UPF corporations were identified. For the full list of corporate interest group and their characteristics, including year founded, headquarter location (city and country), World Bank income level and region, and category type, see supplementary Table 2. The top 50 UPF manufacturers and their total number of interest group connections are shown in Fig. 1.

### The UPF industry’s global corporate influence network

In this section we describe the UPF industry’s corporate influence network by determining which UPF corporations and corporate interest groups were most central to the network, and therefore most likely to coordinate its activities. In total, the most connected UPF corporations (i.e.: with the highest degree centrality) were Nestlé (*n* = 171), The Coca-Cola Company (*n* = 147), Unilever (*n* = 142), PepsiCo (*n* = 138), Danone (*n* = 113), Mars Inc (*n* = 96), Mondelez International Inc (*n* = 89), Ferrero & related parties (*n* = 76), Cargill Inc (*n* = 67), and Bayer (*n* = 66).Just focussing on the 50 leading supply chain and co-dependent industry corporations Bayer (*n* = 66), Reckitt Benckiser (*n* = 59), Abbott (*n* = 55), Royal DSM (*n* = 53), McDonalds (*n* = 51), Walmart (*n* = 41), Syngenta (*n* = 38), and BASF (*n* = 38) were the most connected within the UPF corporate influence network.

**Fig. 2:**
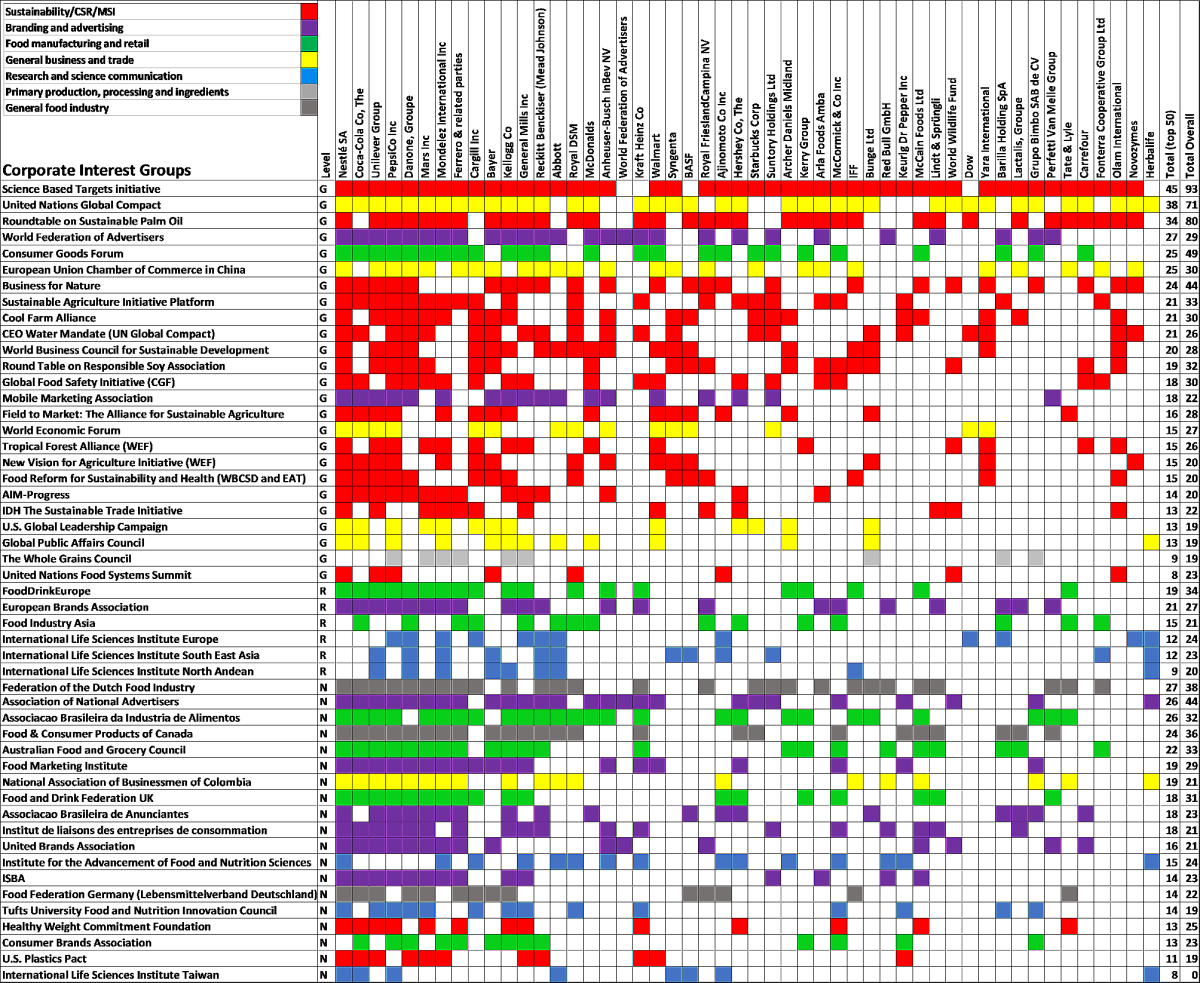
Multi-level grid of the top 50 most connected UPF corporations and corporate influence groups. *Notes* four other corporate interest group types were not included because they were outside the top 50 most connected to the UPF corporations. * The x (left to right) and y-axis (top to bottom) shows the top 50 most connected UPF corporations and corporate interest group actors respectively, in ranked order

Using a multi-level grid, we show the top 50 interest groups (organised by level– global (G), regional (R), national (N)) and type, against the UPF corporations with the largest number of connections, highlighting which UPF corporations hold memberships in each interest group (see Fig. 2). In terms of the corporate interest group memberships and connections with leading UPF corporations, our analysis shows that sustainability label and rating organisations, for example, Science Based Targets initiative (*n* = 93), Roundtable on Sustainable Palm Oil (*n* = 80), the United Nations Global Compact (*n* = 71) and Business for Nature (*n* = 44), are prominent in the UPF corporate influence network. At the national level, branding and advertising corporate interest groups, such as the Association of National Advertisers (ANA), Food Marketing Institute (FMI), Institut de liaisons des entreprises de consommation (Ilec) and the national member advertiser associations of the World Federation of Advertisers (WFA) (e.g.: Associacao Brasileira de Anunciantes, United Brands Association) are those most connected to leading UPF corporations.

**Fig. 3:**
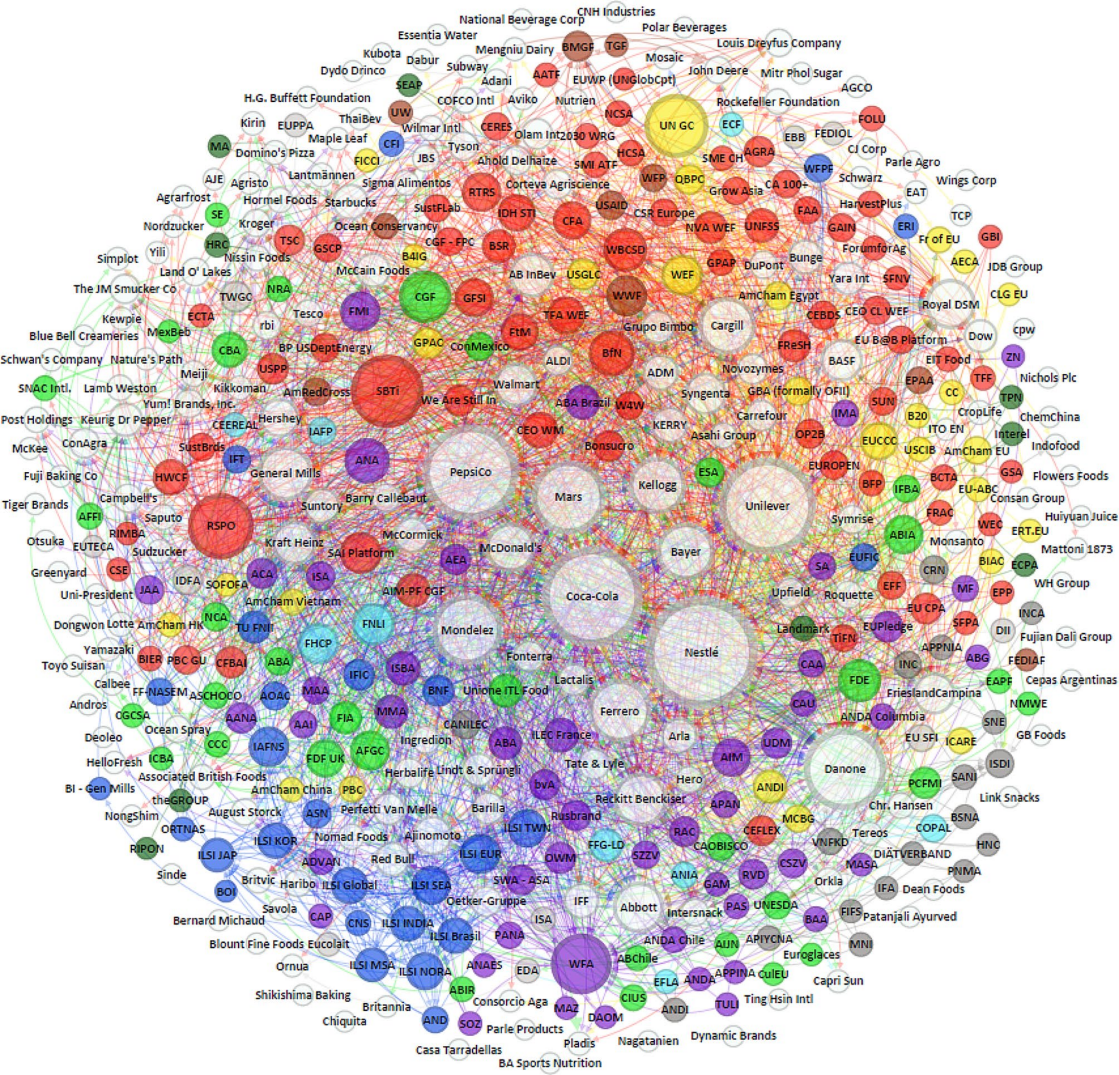
The UPF industry’s global corporate influence network of corporations, organizations and interest groups. Notes The lines represent the links between UPF corporations, food system actors and the UPF system influence network. The circle size is proportionate to the number of ‘links’ the organization has with others in the network. White circles - UPF corporation; Purple circles - Branding and advertising; Yellow circles - General business and trade; Green circles - Food manufacturing and retail; Light grey circles - Primary production, processing and ingredients; Red circles - Sustainability/CSR/MSI; Blue circles - Research and science communication; Brown circles - Other; Dark green circles - Lobbying, legal and public relations; Dark grey circles - Specialized nutrition and baby food; Light blue circles - General food industry. This figure was generated using Gephi version 0.10.1

The UPF industry’s global corporate influence network is represented in Fig. 3. Figure 3 shows that today, sustainability/CSR/MSI interest groups (shown in red) are central to the UPF corporate influence network (*n* = 73, 27.2%), followed by branding and advertising (shown in purple) (*n* = 50, 18.7%), food manufacturing and retail (shown in green) (*n* = 33, 12.3%), general business and trade (shown in yellow) (*n* = 28, 10.4%), and research and science communication interest groups (shown in dark blue) (*n* = 27, 10.1%). The prominance of the red edges or lines and arrows (which indicates connections) of sustainability/CSR/MSI corporate interest groups within the network indicates influence across the corporate influence network. The arrows circling and pointing towards the UPF corporation indicates the types of interest groups with which the corporation engages. The arrows directed at the interest groups indicates membership of interest groups to other interest groups. For example, many branding and advertising interest groups throughout the world are also members of WFA and this is highlighted by the number of purple arrows circling and pointing towards the WFA node. The least prominent according to our classification were both general food industry (shown in dark grey) (*n* = 9, 3.4%) and lobbying, legal and public relations interest groups (shown in dark green) (*n* = 9, 3.4%). The lines represent partnership and/or membership with these organizations and the relative size of the circles represents the total number of organizations with which each network member is associated.

### The corporate interest groups by year founded and type

**Fig. 4:**
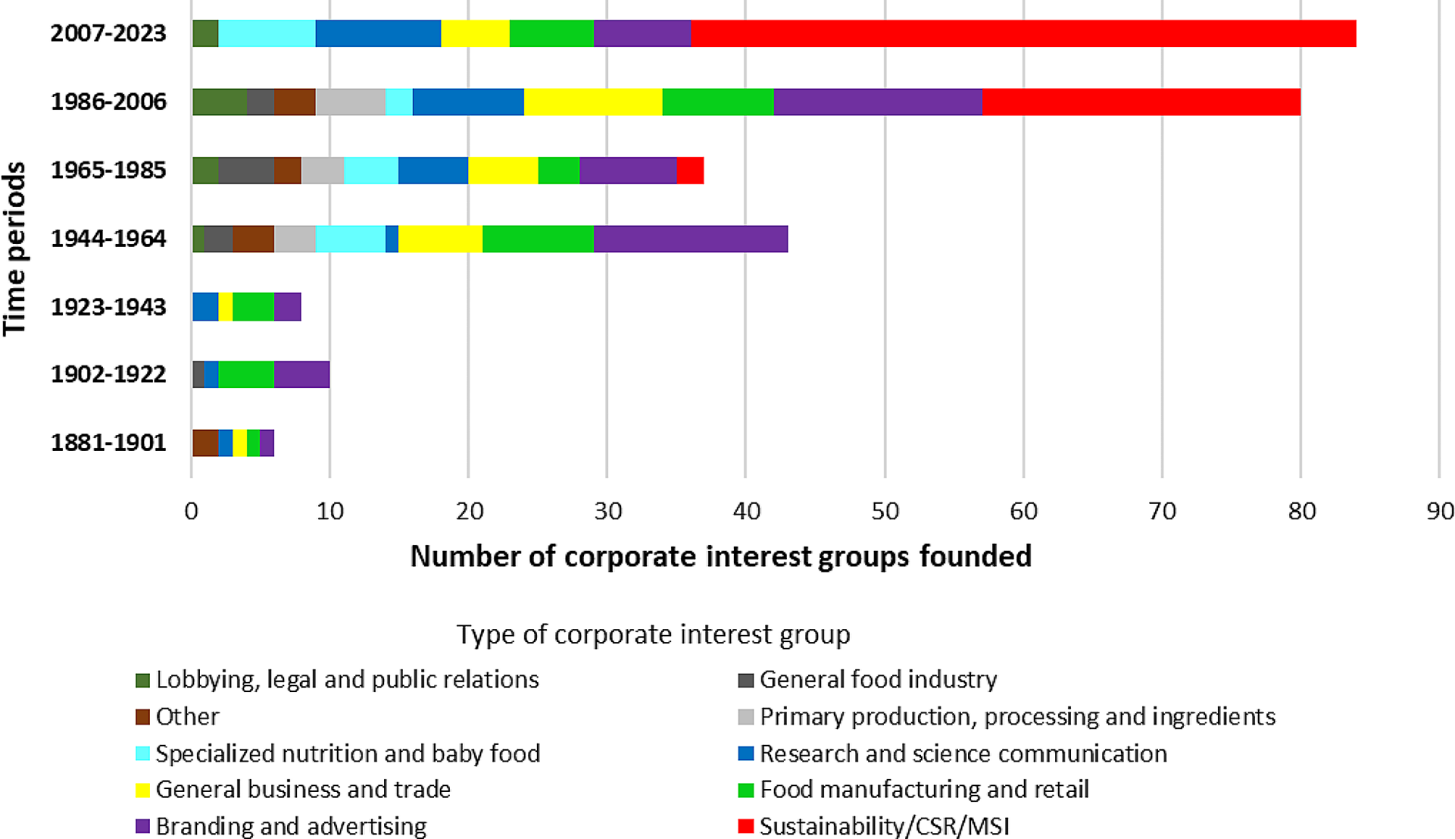
Distribution of corporate interest groups by time-period founded and type

Figure 4 shows the distribution of when the corporate interest groups were founded by 21-year time periods (starting from the when the first interest group was founded) and type. Of note, the most recent period only spans 16 years. There are three distinct time periods noticeable in the data, 1881 to 1943 (*n* = 24), 1944 to 1985 (*n* = 80), and 1986 to present (*n* = 164) with each transition timepoint leading to an increase in corporate interest groups being founded. Within each of those time periods, the largest number of interest groups founded were food manufacturing and retail (*n* = 8/24, 33.3%) in the 1881 to 1943 period; branding and advertising (*n* = 21/80, 26.3%) in the 1944 to 1985 period; and sustainability/CSR/MSI interest groups (*n* = 71/164, 43.3%) in the 1986 to present period, respectively. When organized by level (global, regional, national), at the global level sustainability/CSR/MSI (*n* = 52, 54.2%) and general business and trade interest groups (*n* = 16, 16.7%) are the most prominent.

### Headquarter locations and GFG

**Fig. 5:**
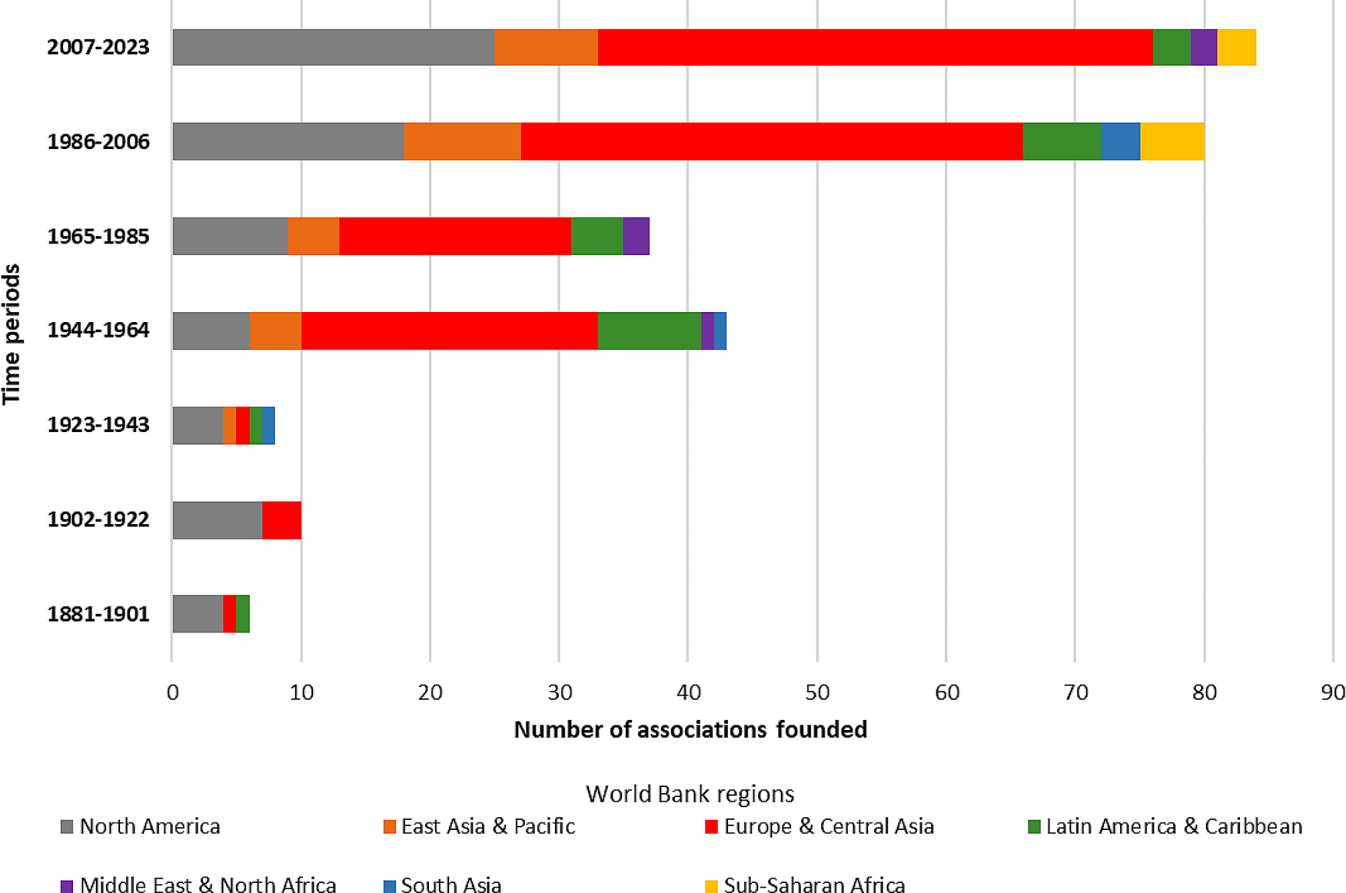
Distribution of corporate interest groups by time-period founded and headquarter location using World Bank regions

**Fig. 6:**
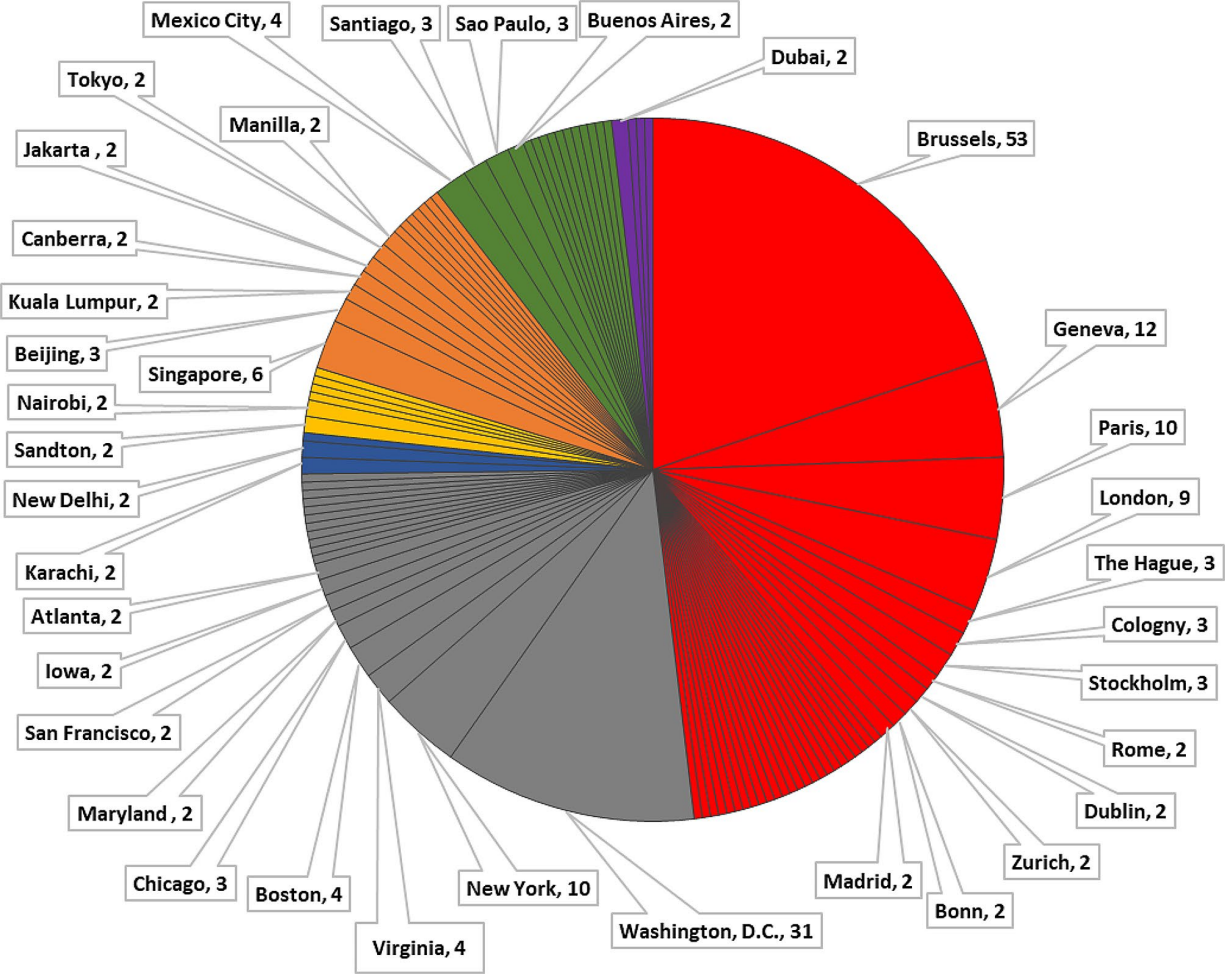
Headquarter city location of corporate interest groups colour coded by World Bank regions. *Notes* Labels in the figure were only included if the city was represented in the data on 2 or more occasions. Orange - East Asia & Pacific; Red - Europe & Central Asia; Green - Latin America & Caribbean; Purple - Middle East & North Africa; Grey– North America; Blue– South Asia; Yellow– Sub-Saharan Africa

The UPF industry as a network found, fund, manage and control [35], and strategically position interest groups headquarters in the food system decision making GFG centres throughout the world. Figure 5 shows that when headquarter location data were organised into regions according to the World Banks regional classifications [61], the region of North America was where most interest groups were founded in the 1881 to 1943 period. In the 1944 to 1985 and 1986 to present period, Europe and Central Asia (Central Asia accounted for 0) was where most interest groups were founded. Combined, North America and Europe accounted for three quarters (75%) of the headquarter locations within the UPF corporate influence network. By country, the United States (*n* = 70/268 or 26.1%), Belgium (*n* = 54/268 or 20.1%), Switzerland (*n* = 20/268 or 7.5%), the United Kingdom (*n* = 12/268 or 4.5%) and France (*n* = 11/268 or 4.1%) were the countries in which most influence network interest groups were headquartered in. When organised and analysed by city (shown in Fig. 6) almost one third (31.3%) were based in either Brussels (*n* = 53/268 or 19.7%) or Washington DC (*n* = 31/268 or 11.6%). When categorised by World Bank country income categories, interest groups were also disproportionately located in high-income countries (*n* = 219/268, 81.7%). Upper-middle-income countries (*n* = 30/268, 11.2%) and lower-middle-income countries (*n* = 19/268, 7.1%) were represented in the headquarter location data, however low-income countries were not (*n* = 0).

## Discussion

This study aimed to map and analyse the UPF industry’s global network of corporate interest groups. Such groups are funded and coordinated by the UPF industry to implement and drive its lobbying and other political activities. Our findings reveal several key features of this global influence network. First, a small number of UPF corporations, including Nestlé, The Coca-Cola Company, Unilever, PepsiCo, Danone, Mars, Mondelez International, and Ferrero are core to the network in terms of most memberships, and are therefore likely to play the leading role in coordinating the UPF industry’s transnational political activities. Second, the corporate interest groups involved in the network are diverse, addressing different regulatory issues and functions across multiple UPF supply chain sectors in the global food system. Third, the network is multi-level, and multi-jurisdictional, spanning global, regional and national levels, with interest groups present in a large number of the UPF industry’s leading country markets. Fourth, the UPF industry’s global influence network has evolved over time, with many interest groups founded from the 1990s onwards, through most recently, an increase in powerful multistakeholder corporate interest groups. These phases coincide with the time periods described in the literature on food regimes, evidenced by large increases in numbers and specific types of interest groups founded over time have occurred. In the section below we discuss the implications of these findings for GFG, food systems transformation, and global policy actions to attenuate the harms of UPFs.

Our analysis suggests that leading global UPF corporations heavily invest resources and effort to build and maintain a network of corporate interest groups. For instance, we found that the top 10 leading UPF corporations have on average 99.5 connections within the identified UPF corporate influence network, with the top 5– PepsiCo, Nestlé, The Coca-Cola Company, Kraft Heinz, and Unilever– averaging 127.8 connections. This high number of membership connections indicates the value these leading UPF corporations give to creating and maintaining an UPF corporate influence network, especially given the total costs which are spent on membership fees [28] in the hope of protecting against ‘business risks’ [62, 63] which may impact profit making. Research suggests that it is not uncommon for leading global UPF corporations to spend tens of millions of dollars in fees to lobby groups or industry associations to support their economic, political, and policy interests [39, 64], for, in return, it is expected that favorable political and policy environments for profiteering are maintained or developed. The cost-benefit of these fees and resources to the UPF industry is clearly worth the investment, considering that the UPF industry’s corporate influence network, has effectively employed tactics to obstruct regulatory policies that may impact profit making abilities [22, 65–67], and has led to UPF corporations becoming some of the largest accumulators of profit and distributors of capital, in the global food system [68].

The existence of a powerful UPF corporate influence network with many functionalities, acting to influence GFG decisions and the global food system activities, raises serious concerns. These concerns relate to both legitimacy issues, and power imbalances and asymmetries that corporate interest group participation creates in specific governance bodies, fora or policy processes by simply having ‘a seat at the table’. This study’s results suggest that the UPF industry’s power and legitimacy is amplified via the UPF industry’s network of corporate interests groups, through the crossover, coverage of, and reach into the multilateral food governance system, UN agencies, and other GFG policy and decision-making spaces. To achieve this, UPF corporations strategically engage a wide range of different corporate interest groups, creating a ‘web-like governance structure’ across key components of global food system, with the intention to create influence systemically. The variation in the different types of corporate interest groups identified attest to this, as leading UPF corporations are highly interconnected at the global, regional, and national level, through common interest group memberships. For example, using the High Level Panel of Experts on Food Security and Nutrition (HLPE) conceptualization of the global food system [69, 70], we see in Fig. 3 that the UPF corporate influence network extends into all components of the system, including the drivers (e.g.: general business and trade; lobbying, legal and public relations), food supply chains (e.g.: primary production, processing and ingredients; general food industry etc.), food environments (e.g.: food manufacturing and retail), consumer behaviors (e.g.: branding and advertising; specialized nutrition and baby food; research and science communication), and policy and governance (e.g.: sustainability/CSR/MSI etc.), an area which was, until recently, predominantly state led and controlled [71]. Indeed, significant crossover due to the interconnectedness of the current global food system exists, however, this impact and coverage across the food system, likely bolsters the growing structural power of major UPF corporate actors, akin to having influence over the ‘rules of the game’. The importance of this with respect to GFG and the food systems transformation agenda according to political economy scholars, is that this type of structural power, confers UPF corporations the ability to ‘circumvent laws and regulations’, to effectively operate ‘above’ the nation state at a ‘supranational level’ [5].

Over time, there have been three distinct increases in the overall number of interest groups within the UPF industry’s corporate influence network, which is consistent with the characteristics of Food Regime Theory proposed by Friedmann and McMichael. Food Regime Theory was first introduced by Friedmann [72] and then described by both in their seminal 1989 paper as: the First - ‘colonial food regime’ (1870–1930 s); the Second - ‘mercantile-industrial food regime’ (1950–1970 s); and the Third -‘corporate-environmental food regime’ (1980s-present) [73]. Literature on food regimes suggests that it offers both a framework which helps us to understand how agriculture has impacted and shaped global development and capitalism [72], and political economy/ecology more broadly [74], in addition to being a methodological tool to structure historical global food system analysis [75]. It is also important to note when discussing food regimes, that the current period has been built on, and redescribed, by McMichael as the ‘corporate food regime’ [76] and Friedmann as ‘green capitalism’ [77]. In explaining the political economy of food regimes, McMichael notes, that although each period provisioned ‘cheap food and food products’ globally, in the case of the ‘industrial’ and ‘corporate’ (second and third) food regimes, a major outcome of these periods has seen food become both ‘reconstituted products’ (i.e., UPFs) and abstracted from its organic relationship with humans [78]. When combined, our results support both McMichael’s and Friedmann’s interpretations, especially when we consider that UPF aligned, sustainability focussed, CSR, multistakeholder interest groups have become the leading ‘support actors’ in GFG, including the multilateral food governance system, for UPF corporations seeking to maintain and further expand UPF systems throughout the world. A recent example of this was the UN Food Systems Summit, where the leadership sought to limit the direct participation of UPF corporations, yet, they were indirectly represented through multistakeholder interest groups and other organizations that are partners and/or have shared interests [79, 80].

We also show through the analysis of headquarter locations, that the US and EU, and more specifically the cities of Washington DC, New York, Brussels, and Geneva are important power centers for the UPF industry’s corporate influence network, suggesting it is potentially where political, economic, and decision-making power in both the global food and UPF system resides. Building this link between the UPF system political economy, global governance structures, and GFG decision making, this finding reinforces how important the founding of (or revitalizing of) several global governance institutions in the mid-1940s (e.g.: World Bank, International Monetary Fund, Food and Agriculture Organization of the United Nations) and the 1990s (e.g.: European Union, World Trade Organization) have been for not only establishing dominant US and EU positions in GFG [81], but for the expansion of both US and EU domiciled UPF transnational food corporations throughout the world. The impact of the these institutions in GFG has been a critical part of a recent IPES-Food report [82] as well as other scholarship, with the premise being that they have ‘a long history of pursuing close collaboration with the corporate sector through industry partnerships’ [83, 84], and the corporate capture of GFG is increasingly taking place in more visible ways; for example, through PPPs [85], the liberalization of trade [86] and the prioritization of policies which aid US and EU businesses [82, 87]. Given that these localities are in which both these key globalist institutions and the UPF corporate influence network are anchored, this finding helps us understand potentially which trade and investment agreements, policies, and partnerships (e.g.: trade liberalization, PPPs) pushed by these institutions to finance and implement ‘development’ and ‘agricultural projects’ [81, 82] around the world, may have also aided the development of UPF systems globally. And thus, in turn, what potential GFG, policy, and trade changes are needed to address the human and ecological health harms of UPF systems.

We found several instances where corporate interest groups have changed their names and this likely has an impact in GFG spaces also. The relevance of this to this study is that many of these groups have strong and direct links with many large UPF TNFCs [88]. For example, Coca-Cola set up and funded a global network of interest groups, the International Life Sciences Institutes (ILSI) for decades, to conduct research and impact nutrition and public health policy [89]. In recent years, ILSI ‘rebranded’ it’s North American organization to the Institute for the Advancement of Food and Nutrition Sciences (IAFNS). Additionally, further investigation shows that ILSI’s Research Foundation (ILSI RF) has also changed its name to Agriculture & Food Systems Institute (AFSI) as a result of being exposed for being a front group for the UPF industry and its supply chain [90]. Another prominent example is the largest food industry association in Washington, DC., the Grocery Manufacturers Association, which rebranded to the Consumer Brands Association [91].

Although it is beyond the scope of this paper to suggest a comprehensive set of prescriptive ideas or recommendations for managing UPF corporate interest group influence, we propose, based on this papers key findings, that reducing UPF industry political power in GFG, could potentially involve actively delegitimizing the UPF industries preferred multi-stakeholder governance model, and particularly those multistakeholder interest groups, which are UPF corporation aligned. Instead, the establishment of an interconnected, cohesive, new global network of institutions and organisations to mobilize national actors, and country specific policies and actions, potentially could offer a platform and opportunity, to initiate the drawing down of UPF production and consumption. Furthermore, the establishment of such a network is supported by recommendations from recent scholarship on the CDOH, where recommendations include the development of ‘new international organisations and institutional arrangements [92], an overarching ‘international regulatory framework’ and ‘rules for engagement and conflicts of interest’ in all international organisations [93], and a ‘health-in-all policies’ approach [19], which potentially could extend to a ‘public health, and healthy and sustainable food systems first approach’ in all policies recommendation. These potential actions combined also call for a strong, cohesive UN position on managing corporate engagement, to create a global, system wide approach, in all GFG spaces.

This paper has several limitations. First, given the analysis focused on the global level, we chose to exclude the influence network of UPF industry actors at the sub-national level as this level of data were only available for 2 of the 10 seed data UPF industry actor websites. Second, our data is likely not an exhaustive representation of all the lobby groups/associations due to the method of data collection which is limited by both the keywords and search terms used and whether the associations chose to disclose members on their publicly available websites and reports. Several examples demonstrate this. One, some UPF industry and corporate influence network members pages were ‘locked’ to only members and required login details to access, particularly those associated with primary production and agri-business. Another example is from a recent study on the Academy of Nutrition and Dietetics, which showed that it has many financial ties to the world’s largest UPF corporations [39], yet we were unable to locate these disclosures, links, or memberships on their website. Third, we acknowledge that this analysis does not provide a complete picture of the entire UPF system as we chose not to include financial actors (i.e., banks, development banks, credit card corporations, accountant firms, investment fund managers etc.), small and medium business, and sub-national and local level actors in the data collection process. However, we recognize that these actors are a major component of the UPF corporate influence network. Finally, we recorded ‘membership’ as it was reported on company websites at the time of data collection, and hence this may not represent actual membership at the time of publication, nor can we validate the accuracy of content sourced from these websites.

## Conclusion

The UPF industry’s corporate influence network is a major structural feature of GFG with strong ties with co-dependent industries to amplify influence. The political power of the UPF industry represents a major challenge for GFG and the health and sustainability food systems transformation agenda. Over the last century and into the present, the UPF industry has formed a powerful, prominent, corporate influence network in global food systems governance, and its strength comes from its globalized nature, strategic positioning, coverage across all components of the food system, scale and supporting corporate ecosystem of actors which all collectively drive UPF systems. These observations have important implications for managing the potential influence of the UPF corporate influence network in political and policy discussions in GFG spaces, especially in attempting to achieve a fairer, healthier, sustainable, and more equitable global food system. Key GFG decision makers attempting to drive systemic change towards this goal must address both UPF corporations and the wider UPF corporate influence network, by positioning them as ‘core to problem’ and not ‘part of the solution’ if healthy and sustainable food systems are to become a reality.

.

### Electronic supplementary material

Below is the link to the electronic supplementary material.

**Supplementary Material 1:** UPF corporations

**Supplementary Material 2:** Associations and lobby groups

## Data Availability

The datasets used and/or analyzed are available from the corresponding author on reasonable request.
