# Supplementary material for: Corporate interest groups and their implications for global food governance: mapping and analysing the global corporate influence network of the transnational ultra-processed food industry
Source: Global Health. 2024 Feb 22;20:16. doi: 10.1186/s12992-024-01020-4 (PMC10882744; doi:10.1186/s12992-024-01020-4)
Supplement: Supplementary file 1 — Supplementary Material 1: UPF corporations [file 12992_2024_1020_MOESM1_ESM.docx]

**Supplementary Table 1:** UPF corporations

| **Corporations** | Nagatanien Co Ltd |
| --- | --- |
| Adani Group | National Beverage Corp |
| Agrarfrost Holding GmbH & Co. KG | Nature's Path Foods Inc |
| Agristo NV | Nestlé SA |
| Aje Group | Nichols Plc |
| Ajinomoto Co Inc | Nissin Foods Holdings Co Ltd |
| Andros SAS | Nomad Foods Ltd |
| Anheuser-Busch InBev NV | Nong Shim Co Ltd |
| Arla Foods Amba | Ocean Spray Cranberries Inc |
| Asahi Group Holdings Ltd | Oetker-Gruppe |
| Associated British Foods Plc | Ornua Co-op Ltd |
| August Storck KG | Otsuka Holdings Co Ltd |
| Aviko Holding B.V | Parle Agro Pvt Ltd |
| BA Sports Nutrition LLC | Parle Products Pvt Ltd |
| Barilla Holding SpA | Patanjali Ayurved Ltd |
| Barry Callebaut | PepsiCo Inc |
| Bernard Michaud SA | Perfetti Van Melle Group |
| Blount Fine Foods Corp | Pladis Ltd |
| Blue Bell Creameries Inc | Polar Beverages |
| Britannia Industries Ltd | Post Holdings Inc |
| Britvic Plc | Red Bull GmbH |
| Bunge Ltd | Royal DSM |
| Calbee Foods Co Ltd | Royal FrieslandCampina NV |
| Campbell Soup Co | Saputo Inc |
| Capri Sun Group Holding AG | Sari Enesis Indah PT |
| Cargill Inc | Savola Group |
| Casa Tarradellas SA | Schwan Food Co, The (Schwan's Company) |
| Cepas Argentinas SA | Shenzhen Eastroc Beverage Co Ltd |
| Cereal Partners Worldwide SA | Shikishima Baking Co Ltd |
| China Huiyuan Juice Group Ltd | Sigma Alimentos SA de CV |
| China Mengniu Dairy Co Ltd | Sinde Budi Sentosa PT |
| China National Cereals, Oils & Foodstuffs Imp & Exp Corp (COFCO) | Starbucks Corp |
| Chiquita Brands International Inc | Suntory Garuda Beverage PT |
| Chocoladefabriken Lindt & Sprüngli AG | Suntory Holdings Ltd |
| CJ Corp | TC Pharmaceutical Industry Co Ltd |
| Coca-Cola Co, The | Thai Beverage PCL |
| Coca-Cola Hellenic Bottling Co SA | Tiandi No1 Beverage Co Ltd |
| ConAgra Brands Inc | Tiger Brands Ltd |
| Consorcio Aga SA de CV | Ting Hsin International Group |
| Dabur India Ltd | Toyo Suisan Kaisha Ltd |
| Danone, Groupe | Tyson Foods Inc |
| Dean Foods Co | Unilever Group |
| Deoleo SA | Uni-President Enterprises Corp |
| Dongwon Group | Upfield Holdings BV |
| Dydo Drinco Inc | Vital Pharmaceuticals Inc (VPX) |
| Dynamic Brands (Pty) Ltd | WH Group |
| Essentia Water LLC | Wilmar International Ltd |
| Ferrero & related parties | Wings Corp |
| Flowers Foods Inc | Yamazaki Baking Co Ltd |
| Fonterra Cooperative Group Ltd | Yangshengtang Co Ltd |
| Fuji Baking Co Ltd | Abbott |
| Fujian Dali Food Co Ltd | Reckitt Benckiser |
| Fürsten-Reform Dr Med Hans Plümer Nachf GmbH & Co | McDonald's |
| GB Foods SA, The | Yum! Brands, Inc. |
| General Mills Inc | Restaurant Brands International Inc |
| Greenyard | Doctor's Associates Inc (Subway) |
| Grupo Bimbo SAB de CV | Domino's Pizza Inc |
| Grupo Lala SAB de CV | International Flavors & Fragrances Inc. |
| Guangdong Jianlibao Group | Tate & Lyle |
| Guangzhou Pharmaceutical Holding Ltd | Novozymes |
| Gujarat Co-operative Milk Marketing Federation Ltd | Kerry Group |
| Haribo GmbH & Co KG | Chr. Hansen |
| HelloFresh SE | Roquette |
| Henan Topfond Original Food Co Ltd | Olam Int |
| Henan Zhongwo Beverage Co Ltd | JBS |
| Hero Group GmbH | Ingredion |
| Hershey Co, The | Symrise |
| Himalaya Food International Ltd. | Dow |
| Hormel Foods Corp | DuPont |
| Idilia Foods SL | Associated British Foods Plc |
| Indofood Sukses Makmur Tbk PT | Bayer |
| Inner Mongolia Yili Industrial Group Co Ltd | Monsanto |
| Intersnack Group GmbH & Co KG | Syngenta |
| ITO EN Ltd | ChemChina |
| JDB Group | BASF |
| Jiaozuo Mingren Natural Medicine Co Ltd | Corteva Agriscience |
| JM Smucker Co, The | Nutrien Ltd |
| Jumex SA de CV, Grupo | The Mosaic Company |
| Kellogg Co | Yara Int |
| Keurig Dr Pepper Inc | John Deere |
| Kewpie Corp | CNH Industries |
| Kikkoman Corp | AGCO |
| Kino Indonesia Tbk PT | Kubota |
| Kirin Holdings Co Ltd | Archer Daniels Midland |
| Kofola CeskoSlovensko as | Louis Dreyfus Company |
| Kraft Heinz Co | Walmart |
| Lactalis, Groupe | Schwarz Group |
| Lamb Weston Holdings, Inc. | Kroger |
| Land O' Lakes Inc | Carrefour |
| Lantmännen ek för | Ahold Delhaize |
| Link Snacks Inc | Aldi |
| Lotte Group | Tesco |
| Maple Leaf Foods Inc | CropLife |
| Mars Inc | Herbalife |
| Mattoni 1873 as | Sudzucker AG |
| McCain Foods Ltd | Consan Group |
| McCormick & Co Inc | Tereos |
| McKee Foods Corp | Nordzucker |
| Reckitt Benckiser (Mead Johnson) | Mitr Phol Sugar Corporation Ltd. |
| Meiji Holdings Co Ltd | Savola Group |
| Mondelez International Inc |  |
