# Supplementary material for: Corporate interest groups and their implications for global food governance: mapping and analysing the global corporate influence network of the transnational ultra-processed food industry
Source: Global Health. 2024 Feb 22;20:16. doi: 10.1186/s12992-024-01020-4 (PMC10882744; doi:10.1186/s12992-024-01020-4)
Supplement: Supplementary file 2 — Supplementary Material 2: Associations and lobby groups [file 12992_2024_1020_MOESM2_ESM.docx]

**Supplementary Table 2:** Associations and lobby groups

| **Name** | **Founded** | **Label** | **City** | **Country** | **WB Income level** | **WB Region** | **Type of association** |
| --- | --- | --- | --- | --- | --- | --- | --- |
| 2030 Water Resources Group (World Bank) | 2008 | 2030 WRG | Washington, D.C. | USA | High Income | North America | Sustainability/CSR/MSI |
| Academy of Nutrition and Dietetics eatright.org | 1917 | AND | Chicago | USA | High Income | North America | Research and science communication |
| Advertisers Association of Nigeria | 1992 | ADVAN | Ikeja-Lagos | Nigeria | Lower middle income | Sub-Saharan Africa | Branding and advertising |
| Advertisers Association Turkey | 1992 | RVD | Istanbul | Turkey | Upper middle income | Europe & Central Asia | Branding and advertising |
| Advertising Business Group | 2016 | ABG | Dubai | United Arab Emirates | High income | Middle East & North Africa | Branding and advertising |
| African Agricultural Technology Foundation | 2003 | AATF | Nairobi | Kenya | Lower middle income | Sub-Saharan Africa | Sustainability/CSR/MSI |
| AIM-Progress | 2007 | AIM-PF CGF | Brussels | Belgium | High income | Europe & Central Asia | Sustainability/CSR/MSI |
| Alliance for a Green Revolution in Africa | 2006 | AGRA | Nairobi | Kenya | Lower middle income | Sub-Saharan Africa | Sustainability/CSR/MSI |
| Alliance of CEO Climate Leaders (WEF) | 2014 | CEO Climate | Geneva | Switzerland | High income | Europe & Central Asia | Sustainability/CSR/MSI |
| American Beverage Association | 1919 | ABA | Washington, D.C. | USA | High income | North America | Food manufacturing and retail |
| American Chamber of Commerce Egypt | 1982 | AmCham Egypt | Giza | Egypt | Lower middle income | Middle East & North Africa | General business and trade |
| American Chamber of Commerce in China | 1991 | AmCham China | Beijing | China | Upper middle income | East Asia & Pacific | General business and trade |
| American Chamber of Commerce in Hong Kong | 1969 | AmCham HK | Hong Kong Central | Hong Kong | High income | East Asia & Pacific | General business and trade |
| American Chamber of Commerce to the European Union | 1948 | AmCham EU | Brussels | Belgium | High income | Europe & Central Asia | General business and trade |
| American European Community Association | 1981 | AECA | Brussels | Belgium | High income | Europe & Central Asia | General business and trade |
| American Frozen Food Institute | 1942 | AFFI | Virginia | USA | High income | North America | Food manufacturing and retail |
| American Red Cross | 1881 | AmRedCross | Washington, D.C. | USA | High income | North America | Other |
| American Society for Nutrition | 1928 | ASN | Maryland | USA | High income | North America | Research and science communication |
| AOAC International | 1884 | AOAC | Maryland | USA | High income | North America | Research and science communication |
| Asia Pacific Infant and Young Child Nutrition Association | 2010 | APIYCNA | Singapore | Singapore | High income | East Asia & Pacific | Specialized nutrition and baby food |
| Asociacion Espanola de Anunciantes | 1965 | AEA | Madrid | Spain | High income | Europe & Central Asia | Branding and advertising |
| Asociacion Mexicana de Bebidas | 1945 | MexBeb | Mexico City | Mexico | Upper middle income | Latin America & Caribbean | Food manufacturing and retail |
| Asociacion Nacional de Anunciantes de Bolivia | 2012 | ANDA | La Paz | Bolivia | Lower middle income | Latin America & Caribbean | Branding and advertising |
| Asociacion Nacional de Anunciantes de Colombia | 1979 | ANDA Columbia | Bogata | Colombia | Upper middle income | Latin America & Caribbean | Branding and advertising |
| Asociacion Nacional de Anunciantes de El Salvador | 1966 | ANAES | San Salvador | El Salvador | Lower middle income | Latin America & Caribbean | Branding and advertising |
| Asociacion Nacional de Avisadores de Chile | 1964 | ANDA Chile | Vitacura | Chile | High income | Latin America & Caribbean | Branding and advertising |
| Asociacion Nacional de Fabricantes de Chocolates, Dulces y Similares A.C. | 1936 | ASCHOCO | Mexico City | Mexico | Upper middle income | Latin America & Caribbean | Food manufacturing and retail |
| Asociación Nacional de fabricantes de productos de Dietética Infantil | 1977 | ANDI | Madrid | Spain | High income | Europe & Central Asia | Specialized nutrition and baby food |
| Asosiasi Perusahaan Pengiklan Indonesia | 2005 | APPINA | Jakarta | Indonesia | Lower middle income | East Asia & Pacific | Branding and advertising |
| Associacao Brasileira da Industria de Alimentos (Brazilian Food Industry Association) | 2002 | ABIA | Sao Paulo | Brazil | Upper middle income | Latin America & Caribbean | Food manufacturing and retail |
| Associacao Brasileira das Industrias de Refrigerantes e de Bebidas nao Alcoolicas | 1950 | ABIR | Brasilia | Brazil | Upper middle income | Latin America & Caribbean | Food manufacturing and retail |
| Associacao Brasileira de Anunciantes | 1959 | ABA Brazil | Sao Paulo | Brazil | Upper middle income | Latin America & Caribbean | Branding and advertising |
| Associacao Portuguesa de Anunciantes | 1987 | APAN | Lisbon | Portugal | High income | Europe & Central Asia | Branding and advertising |
| Association Nationale des Industries Alimentaires | 1968 | ANIA | Paris | France | High income | Europe & Central Asia | General food industry |
| Association of Advertisers in Ireland | 2002 | AAI | Dublin | Ireland | High income | Europe & Central Asia | Branding and advertising |
| Association of Canadian Advertisers | 1914 | ACA | Montreal | Canada | High income | North America | Branding and advertising |
| Association of Chocolate, Biscuit and Confectionery Industries of Europe | 1959 | CAOBISCO | Brussels | Belgium | High income | Europe & Central Asia | Food manufacturing and retail |
| Association of Dutch Manufacturers of Children's and Diet Foods | 1961 | VNFKD | The Hague | The Netherlands | High income | Europe & Central Asia | Specialized nutrition and baby food |
| Association of National Advertisers | 1910 | ANA | New York | USA | High Income | North America | Branding and advertising |
| Association of Nutritious Products for Mothers and Children Companies | 1992 | APPNIA | Jakarta | Indonesia | Lower middle income | East Asia & Pacific | Specialized nutrition and baby food |
| Association of Swiss Advertisers | 1950 | SWA - ASA | Zurich | Switzerland | High income | Europe & Central Asia | Branding and advertising |
| Australian Association of National Advertisers | 1928 | AANA | Sydney | Australia | High income | East Asia & Pacific | Branding and advertising |
| Australian Food and Grocery Council | 1995 | AFGC | Canberra | Australia | High income | East Asia & Pacific | Food manufacturing and retail |
| B20 (Business at the G20) | 2010 | B20 | Paris | France | High income | Europe & Central Asia | General business and trade |
| Bell Institute of Health and Nutrition | 1963 | BI-GEN MILLS | Minneapolis | USA | High income | North America | Research and science communication |
| Benioff Ocean Initiative | 2016 | BOI | Santa Barbara | USA | High income | North America | Research and science communication |
| Better Plants U.S. Department of Energy | 2011 | BP USDeptEnergy | Washington, D.C. | USA | High income | North America | Sustainability/CSR/MSI |
| Beverage Industry Environmental Roundtable | 2006 | BIER | Mississippi | USA | High income | North America | Sustainability/CSR/MSI |
| Bill & Melinda Gates Foundation | 2000 | BMGF | Seattle | USA | High income | North America | Other |
| Bonsucro | 2005 | Bonsucro | London | UK | High income | Europe & Central Asia | Sustainability/CSR/MSI |
| British Nutrition Foundation | 1967 | BNF | London | UK | High income | Europe & Central Asia | Research and science communication |
| British Specialist Nutrition Association | 2007 | BSNA | London | UK | High income | Europe & Central Asia | Specialized nutrition and baby food |
| Bulgarian Association of Advertisers | 2010 | BAA | Sofia | Bulgaria | Upper middle income | Europe & Central Asia | Branding and advertising |
| Business and Industry Advisory Committee to the OECD | 1962 | BIAC | Paris | France | High income | Europe & Central Asia | General business and trade |
| Business Call to Action | 2008 | BCTA | Istanbul | Turkey | Upper middle income | Europe & Central Asia | Sustainability/CSR/MSI |
| Business Fights Poverty | 2006 | BFP | London | UK | High income | Europe & Central Asia | Sustainability/CSR/MSI |
| Business for Inclusive Growth (OECD) | 2019 | B4IG | Paris | France | High income | Europe & Central Asia | General business and trade |
| Business for Nature | 2019 | BfN | New York | USA | High income | North America | Sustainability/CSR/MSI |
| Business for Social Responsibility | 1992 | BSR | San Francisco | USA | High income | North America | Sustainability/CSR/MSI |
| bvA netwerk van merkleiders | 1919 | bvA | Amsterdam | The Netherlands | High income | Europe & Central Asia | Branding and advertising |
| Calorie Control Council | 1966 | CCC | Atlanta | USA | High income | North America | Food manufacturing and retail |
| Camara Argentina de Anunciantes | 1959 | CAA | Buenos Aires | Argentina | Upper middle income | Latin America & Caribbean | Branding and advertising |
| Camara de Anunciantes del Paraguay | 1997 | CAP | Santa Rosa | Paraguay | Upper middle income | Latin America & Caribbean | Branding and advertising |
| Camara de Anunciantes del Uruguay | 1981 | CAU | Montevideo | Uruguay | High income | Latin America & Caribbean | Branding and advertising |
| Camara Nacional de industriales de la leche | 1961 | CANILEC | Mexico City | Mexico | Upper middle income | Latin America & Caribbean | Specialized nutrition and baby food |
| Canadian Nutrition Society | 2010 | CNS | Ottawa | Canada | High income | North America | Research and science communication |
| Centre for Sustainability and Excellence | 2004 | CSE | Chicago | USA | High income | North America | Sustainability/CSR/MSI |
| CEO Water Mandate (UN Global Compact) | 2020 | CEO WM | New York | USA | High income | North America | Sustainability/CSR/MSI |
| Ceres | 1989 | CERES | Boston | USA | High income | North America | Sustainability/CSR/MSI |
| Chamber of Food Manufacturers Inc. - Philippines | 1958 | PCFMI | Manilla | Philippines | Lower middle income | East Asia & Pacific | Food manufacturing and retail |
| Children’s Food and Beverage Advertising Initiative | 2007 | CFBAI | New York | USA | High income | North America | Sustainability/CSR/MSI |
| Circular Economy for Flexible Packaging | 2016 | CEFLEX | LA Bergschenhoek | The Netherlands | High income | Europe & Central Asia | Sustainability/CSR/MSI |
| Circulate Capital | 2018 | CC | New York | USA | High income | North America | General business and trade |
| Climate Action 100+ | 2017 | CA 100+ | Boston | USA | High income | North America | Sustainability/CSR/MSI |
| Committee of European Sugar Users | 1995 | CIUS | Brussels | Belgium | High income | Europe & Central Asia | Food manufacturing and retail |
| Conselho Empresarial Brasileiro para o Desenvolvimento Sustentável | 1997 | CEBDS | Rio de Janeiro | Brazil | Upper middle income | Latin America & Caribbean | Sustainability/CSR/MSI |
| Consumer Brands Association | 1908 | CBA | Washington, D.C. | USA | High income | North America | Food manufacturing and retail |
| Consumer Goods Council of South Africa | 2002 | CGCSA | Sandton | South Africa | Upper middle income | Sub-Saharan Africa | Food manufacturing and retail |
| Consumer Goods Forum | 2009 | CGF | Paris | France | High income | Europe & Central Asia | Food manufacturing and retail |
| Cool Farm Alliance | 2008 | CFA | Lincolnshire | UK | High income | Europe & Central Asia | Sustainability/CSR/MSI |
| Corporate Leaders Group Europe | 2005 | CLG EU | Cambridge | UK | High income | Europe & Central Asia | General business and trade |
| Council for Responsible Nutrition | 1973 | CRN | Washington, D.C. | USA | High income | North America | Specialized nutrition and baby food |
| Culinaria Europe | 1959 | CulEU | Bonn | Germany | High income | Europe & Central Asia | Food manufacturing and retail |
| Czech Association for Branded Products | 1993 | CSZV | Prague | Czech Republic | High income | Europe & Central Asia | Branding and advertising |
| Dairy Industry Ireland | 1993 | DII | Dublin | Ireland | High income | Europe & Central Asia | Primary production, processing and ingredients |
| Danske Annoncører og Markedsførere | 2020 | DAOM | Copenhagen | Denmark | High income | Europe & Central Asia | Branding and advertising |
| EIT Food | 2016 | EIT Food | Brussels | Belgium | High income | Europe & Central Asia | Sustainability/CSR/MSI |
| Estonian Marketing Association - Turundajate Liit | 2013 | TULI | Tallinn | Estonia | High income | Europe & Central Asia | Branding and advertising |
| EU Pledge | 2007 | EUPledge | Brussels | Belgium | High income | Europe & Central Asia | Branding and advertising |
| EU Specialty Food Ingredients | 1983 | EU SFI | Brussels | Belgium | High income | Europe & Central Asia | Primary production, processing and ingredients |
| EU Vegetable Oil and Proteinmeal Industry Association | 1958 | FEDIOL | Brussels | Belgium | High income | Europe & Central Asia | Primary production, processing and ingredients |
| EU Circular Plastics Alliance | 2019 | EU CPA | Brussels | Belgium | High income | Europe & Central Asia | Sustainability/CSR/MSI |
| EU-ASEAN Business Council | 2011 | EU-ABC | Singapore | Singapore | High income | East Asia & Pacific | General business and trade |
| Euroglaces Ice Cream Association | 1961 | Euroglaces | Brussels | Belgium | High income | Europe & Central Asia | Food manufacturing and retail |
| European Alliance for Plant-based Foods | 2020 | EAPF | Brussels | Belgium | High income | Europe & Central Asia | Food manufacturing and retail |
| European Association of Dairy Trade | 1959 | Eucolait | Brussels | Belgium | High income | Europe & Central Asia | Primary production, processing and ingredients |
| European Biodiesel Board | 1997 | EBB | Brussels | Belgium | High income | Europe & Central Asia | Primary production, processing and ingredients |
| European Brands Association | 1967 | AIM | Brussels | Belgium | High income | Europe & Central Asia | Branding and advertising |
| European Breakfast Cereal Association | 1992 | CEEREAL | Brussels | Belgium | High income | Europe & Central Asia | General food industry |
| European Centre of Public Affairs | 1986 | ECPA | Brussels | Belgium | High income | Europe & Central Asia | Lobbying, legal and public relations |
| European Clean Trucking Alliance | 2020 | ECTA | The Hague | Netherlands | High income | Europe & Central Asia | Sustainability/CSR/MSI |
| European Coffee Federation | 1981 | ECF | Brussels | Belgium | High income | Europe & Central Asia | General food industry |
| European Commission's Business and Biodiversity Platform | 2008 | EU B@B Platform | Brussels | Belgium | High income | Europe & Central Asia | Sustainability/CSR/MSI |
| European Dairy Association | 1995 | EDA | Brussels | Belgium | High income | Europe & Central Asia | Primary production, processing and ingredients |
| European Food Forum | 2019 | EFF | Brussels | Belgium | High income | Europe & Central Asia | Sustainability/CSR/MSI |
| European Food Information Council | 1995 | EUFIC | Brussels | Belgium | High income | Europe & Central Asia | Research and science communication |
| European Food Law Association | 1973 | EFLA | Brussels | Belgium | High income | Europe & Central Asia | General food industry |
| European Fruit Juice Association | 1962 | AIJN | Brussels | Belgium | High income | Europe & Central Asia | Food manufacturing and retail |
| European Organization for Packaging and the Environment | 1991 | EUROPEN | Etterbeek | Belgium | High income | Europe & Central Asia | Sustainability/CSR/MSI |
| European Partnership for Alternative Approaches to Animal Testing | 2005 | EPAA | Brussels | Belgium | High income | Europe & Central Asia | Other |
| European Pet Food Industry | 1970 | FEDIAF | Brussels | Belgium | High income | Europe & Central Asia | Other |
| European Plastics Pact | 2020 | EPP | Brussels | Belgium | High income | Europe & Central Asia | Sustainability/CSR/MSI |
| European Potato Processors' Association | 1962 | EUPPA | Brussels | Belgium | High income | Europe & Central Asia | Primary production, processing and ingredients |
| European Roundtable of Industrialists | 1983 | ERT.EU | Brussels | Belgium | High income | Europe & Central Asia | General business and trade |
| European Snacks Association | 1965 | ESA | Brussels | Belgium | High income | Europe & Central Asia | Food manufacturing and retail |
| European Technical Caramel Association | 1978 | EUTECA | Brussels | Belgium | High income | Europe & Central Asia | Primary production, processing and ingredients |
| European Union Chamber of Commerce in China | 2000 | EUCCC | Beijing | China | Upper middle income | East Asia & Pacific | General business and trade |
| European Water Partnership (UN Global Compact) | 2006 | EUWP (UNGlobCpt) | Brussels | Belgium | High income | Europe & Central Asia | Sustainability/CSR/MSI |
| Exponential Roadmap Initiative | 2018 | ERI | Stockholm | Sweden | High income | Europe & Central Asia | Research and science communication |
| Federal Association of Special Foods | 1948 | DIÄTVERBAND | Bonn | Germany | High income | Europe & Central Asia | Specialized nutrition and baby food |
| Federation of Indian Chambers of Commerce & Industry | 1927 | FICCI | New Delhi | India | Lower middle income | South Asia | General business and trade |
| Federation of the Dutch Food Industry | 2004 | FNLI | The Hague | The Netherlands | High income | Europe & Central Asia | General food industry |
| Field to Market: The Alliance for Sustainable Agriculture | 2007 | FtM | Washington D.C. | USA | High income | North America | Sustainability/CSR/MSI |
| Food & Consumer Products of Canada | 1959 | FHCP | Ontario | Canada | High income | North America | General food industry |
| Food Action Alliance (WEF) | 2019 | FAA | Cologny | Switzerland | High income | Europe & Central Asia | Sustainability/CSR/MSI |
| Food and Drink Federation UK | 1913 | FDF UK | London | UK | High income | Europe & Central Asia | Food manufacturing and retail |
| Food and Drinks of Chile AG - ABChile | 2014 | ABChile | Santiago | Chile | High income | Latin America & Caribbean | Food manufacturing and retail |
| Food and Land Use Coalition | 2017 | FOLU | London | UK | High income | Europe & Central Asia | Sustainability/CSR/MSI |
| Food Federation Germany (Lebensmittelverband Deutschland) | 1955 | FFG-LD | Nuremberg | Germany | High income | Europe & Central Asia | General food industry |
| Food Forum (National Academy of Sciences) | 1993 | FF-NASEM | Washington, D.C. | USA | High income | North America | Research and science communication |
| Food Industry Asia | 2010 | FIA | Singapore | Singapore | High income | East Asia & Pacific | Food manufacturing and retail |
| Food Marketing Institute | 1976 | FMI | Virginia | USA | High income | North America | Branding and advertising |
| Food Reform for Sustainability and Health (WBCSD and EAT Partnership) | 2017 | FReSH | Geneva | Switzerland | High income | Europe & Central Asia | Sustainability/CSR/MSI |
| Food Research & Action Center | 1970 | FRAC | Washington, D.C. | USA | High income | North America | Sustainability/CSR/MSI |
| FoodDrinkEurope | 1982 | FDE | Brussels | Belgium | High income | Europe & Central Asia | Food manufacturing and retail |
| Forest Positive Coalition (CGF) | 2020 | CGF - FPC | Paris | France | High income | Europe & Central Asia | Sustainability/CSR/MSI |
| Forum for the Future of Agriculture | 2008 | ForumforAg | Brussels | Belgium | High income | Europe & Central Asia | Sustainability/CSR/MSI |
| French Infant Food Sector | 1949 | FIFS | Paris | France | High income | Europe & Central Asia | Specialized nutrition and baby food |
| Friends of Europe | 1999 | Fr of EU | Brussels | Belgium | High income | Europe & Central Asia | General business and trade |
| Global Alliance for Improved Nutrition | 2002 | GAIN | Geneva | Switzerland | High income | Europe & Central Asia | Sustainability/CSR/MSI |
| Global Business Initiative on Human Rights | 2009 | GBI | East Sussex | UK | High income | Europe & Central Asia | Sustainability/CSR/MSI |
| Global Food Safety Initiative (CGF) | 2000 | GFSI | Levallois-Perret | France | High income | Europe & Central Asia | Sustainability/CSR/MSI |
| Global Plastic Action Partnership | 2018 | GPAP | Cologny | Switzerland | High income | Europe & Central Asia | Sustainability/CSR/MSI |
| Global Public Affairs Council | 1954 | GPAC | Brussels | Belgium | High income | Europe & Central Asia | General business and trade |
| Global Shea Alliance | 2011 | GSA | Accra | Ghana | Lower middle income | Sub-Saharan Africa | Sustainability/CSR/MSI |
| Global Social Compliance Programme (CGF) | 2006 | GSCP | Paris | France | High income | Europe & Central Asia | Sustainability/CSR/MSI |
| Groupement des Annonceurs du Maroc | 1984 | GAM | Casablanca | Morocco | Lower middle income | Middle East & North Africa | Branding and advertising |
| Grow Asia | 2015 | Grow Asia | Singapore | Singapore | High income | East Asia & Pacific | Sustainability/CSR/MSI |
| HarvestPlus | 2004 | HarvestPlus | Washington D.C. | USA | High income | North America | Sustainability/CSR/MSI |
| Healthcare Nutrition Council | 2016 | HNC | Washington, D.C. | USA | High income | North America | Specialized nutrition and baby food |
| Healthy Weight Commitment Foundation | 2009 | HWCF | Washington, D.C. | USA | High income | North America | Sustainability/CSR/MSI |
| High Carbon Stock Approach | 2014 | HCSA | Kuala Lumpur | Malaysia | Upper middle income | East Asia & Pacific | Sustainability/CSR/MSI |
| Human Rights Campaign | 1980 | HRC | Washington, D.C. | USA | High income | North America | Lobbying, legal and public relations |
| IDH The Sustainable Trade Initiative | 2008 | IDH STI | Utrecht | The Netherlands | High income | Europe & Central Asia | Sustainability/CSR/MSI |
| Infant Feeding Association of South Africa | 2010 | IFA | Johannesburg | South Africa | Upper middle income | Sub-Saharan Africa | Specialized nutrition and baby food |
| Infant Nutrition Council (Australia and New Zealand) | 2009 | INC | Canberra | Australia | High income | East Asia & Pacific | Specialized nutrition and baby food |
| Infant Nutrition Council of America | 2009 | INCA | Atlanta | USA | High income | North America | Specialized nutrition and baby food |
| Institut de liaisons des entreprises de consommation | 1959 | ILEC France | Paris | France | High income | Europe & Central Asia | Branding and advertising |
| Institute for the Advancement of Food and Nutrition Sciences (formerly ILSI) | 2021 | IAFNS | Washington, D.C. | USA | High income | North America | Research and science communication |
| Institute of Food Technologists | 1939 | IFT | Chicago | USA | High income | North America | Research and science communication |
| Instituto Chileno de Administración Racional de Empresas | 1953 | ICARE | Santiago | Chile | High income | Latin America & Caribbean | General business and trade |
| Interel European Affairs | 1983 | Interel | Brussels | Belgium | High income | Europe & Central Asia | Lobbying, legal and public relations |
| International Association for Food Protection | 1911 | IAFP | Iowa | USA | High income | North America | General food industry |
| International Council of Beverage Associations | 1995 | ICBA | Washington, D.C. | USA | High income | North America | Food manufacturing and retail |
| International Dairy Foods Association | 1989 | IDFA | Washington, D.C. | USA | High income | North America | Primary production, processing and ingredients |
| International Food & Beverage Alliance | 2008 | IFBA | Geneva | Switzerland | High income | Europe & Central Asia | Food manufacturing and retail |
| International Food Information Council | 1985 | IFIC | Washington, D.C. | USA | High income | North America | Research and science communication |
| International Life Sciences Institute Brazil | 1990 | ILSI Brazil | Sao Paulo | Brazil | Upper middle income | Latin America & Caribbean | Research and science communication |
| International Life Sciences Institute Europe | 1986 | ILSI EUR | Brussels | Belgium | High income | Europe & Central Asia | Research and science communication |
| International Life Sciences Institute Global | 1978 | ILSI Global | Washington, D.C. | USA | High income | North America | Research and science communication |
| International Life Sciences Institute India | 1997 | ILSI INDIA | New Delhi | India | Lower middle income | South Asia | Research and science communication |
| International Life Sciences Institute Japan | 1981 | ILSI JAP | Tokyo | Japan | High income | East Asia & Pacific | Research and science communication |
| International Life Sciences Institute Korea | 1981 | ILSI KOR | Seoul | Korea | High income | East Asia & Pacific | Research and science communication |
| International Life Sciences Institute Mesoamerica | 2014 | ILSI MSA | Heredia | Costa Rica | Upper middle income | Latin America & Caribbean | Research and science communication |
| International Life Sciences Institute North Andean | 1997 | ILSI NORA | Bogota | Colombia | Upper middle income | Latin America & Caribbean | Research and science communication |
| International Life Sciences Institute Southeast Asia | 1993 | ILSI SEA | Singapore | Singapore | High income | East Asia & Pacific | Research and science communication |
| International Life Sciences Institute Taiwan | 2013 | ILSI TWN | Taipei City | Taiwan | High income | East Asia & Pacific | Research and science communication |
| International Special Dietary Foods Industries | 1965 | ISDI | Brussels | Belgium | High income | Europe & Central Asia | Specialized nutrition and baby food |
| International Sweeteners Association | 1983 | ISA | Brussels | Belgium | High income | Europe & Central Asia | Primary production, processing and ingredients |
| ISBA | 1891 | ISBA | London | UK | High income | Europe & Central Asia | Branding and advertising |
| Israel Marketing Association | 1961 | IMA | Tel Aviv | Israel | High income | Middle East & North Africa | Branding and advertising |
| Japan Advertisers Association | 1957 | JAA | Tokyo | Japan | High income | East Asia & Pacific | Branding and advertising |
| Landmark Public Affairs | 2007 | Landmark | Brussels | Belgium | High income | Europe & Central Asia | Lobbying, legal and public relations |
| Malaysian Advertisers Association | 1964 | MAA | Selangor | Malaysia | Upper middle income | East Asia & Pacific | Branding and advertising |
| Marketers Association of Zimbabwe | 2007 | MAZ | Harare | Zimbabwe | Lower middle income | Sub-Saharan Africa | Branding and advertising |
| Marketing Association of South Africa | 2006 | MASA | Sandton | South Africa | Upper middle income | Sub-Saharan Africa | Branding and advertising |
| Marketing Finland | 1951 | MF | Helsinki | Finland | High income | Europe & Central Asia | Branding and advertising |
| Medical Nutrition International Industry | 2005 | MNI | Brussels | Belgium | High income | Europe & Central Asia | Specialized nutrition and baby food |
| Mexican Council of the Consumer Products Industry AC | 1996 | ConMexico | Mexico City | Mexico | Upper middle income | Latin America & Caribbean | Food manufacturing and retail |
| Mobile Marketing Association | 2000 | MMA | New York | USA | High income | North America | Branding and advertising |
| Monument Advocacy | 2006 | MA | Washington, D.C. | USA | High income | North America | Lobbying, legal and public relations |
| Multinational Companies Business Group | 2014 | MCBG | Dubai | United Arab Emirates | High income | Middle East & North Africa | General business and trade |
| National Association of Businessmen of Colombia | 1944 | ANDI | Medellín | Colombia | Upper middle income | Latin America & Caribbean | General business and trade |
| National Confectioners Association | 1884 | NCA | Washington, D.C. | USA | High income | North America | Food manufacturing and retail |
| National Restaurant Association | 1919 | NRA | Washington, D.C. | USA | High income | North America | Food manufacturing and retail |
| Natural Climate Solutions Alliance (WBCSD) | 2019 | NCSA | Geneva | Switzerland | High income | Europe & Central Asia | Sustainability/CSR/MSI |
| Natural Mineral Waters Europe | 2003 | NMWE | Brussels | Belgium | High income | Europe & Central Asia | Food manufacturing and retail |
| New Vision for Agriculture Initiative (WEF) | 2009 | NVA WEF | Cologny | Switzerland | High income | Europe & Central Asia | Sustainability/CSR/MSI |
| Obesity Round Table (National Academy of Sciences) | 2014 | ORTNAS | Washington, D.C. | USA | High income | North America | Research and science communication |
| Ocean Conservancy | 1972 | OceanConsr-vy | Washington, D.C. | USA | High income | North America | Other |
| One Planet Business for Biodiversity (WBCSD) | 2019 | OP2B | Geneva | Switzerland | High income | Europe & Central Asia | Sustainability/CSR/MSI |
| Organization for International Investment Inc. (Global Business Alliance) | 1990 | GBA (formally OFII) | Washington, D.C. | USA | High income | North America | General business and trade |
| Organization Werbungtreibende im Markenverband | 1995 | OWM | Berlin | Germany | High income | Europe & Central Asia | Branding and advertising |
| Pakistan Advertisers Society | 1996 | PAS | Karachi | Pakistan | Lower middle income | South Asia | Branding and advertising |
| Pakistan Business Council | 2005 | PBC | Karachi | Pakistan | Lower middle income | South Asia | General business and trade |
| Pediatric Nutrition Manufacturer Association | 1984 | PNMA | Bangkok | Thailand | Upper middle income | East Asia & Pacific | Specialized nutrition and baby food |
| Philippine Association of National Advertisers | 1958 | PANA | Manila | Philippines | Lower middle income | East Asia & Pacific | Branding and advertising |
| Portion Balance Coalition (Georgetown University) | 2011 | PBC GU | Washington, D.C. | USA | High income | North America | Sustainability/CSR/MSI |
| Quality Brands Protection Committee, China Association of Enterprises with Foreign Investment | 2000 | QBPC | Beijing | China | Upper middle income | East Asia & Pacific | General business and trade |
| Responsible Advertising and Children | 1953 | RAC | Brussels | Belgium | High income | Europe & Central Asia | Branding and advertising |
| Rimba Collective | 2021 | RIMBA | Singapore | Singapore | High income | East Asia & Pacific | Sustainability/CSR/MSI |
| Ripon Society | 1962 | RIPON | Washington, D.C. | USA | High income | North America | Lobbying, legal and public relations |
| Round Table on Responsible Soy Association | 2006 | RTRS | Zurich | Switzerland | High income | Europe & Central Asia | Sustainability/CSR/MSI |
| Roundtable on Sustainable Palm Oil | 2004 | RSPO | Kuala Lumpur | Malaysia | Upper middle income | East Asia & Pacific | Sustainability/CSR/MSI |
| Rusbrand | 2002 | Rusbrand | Moscow | Russia | Upper middle income | Europe & Central Asia | Branding and advertising |
| Scaling Up Nutrition | 2010 | SUN | Geneva | Switzerland | High income | Europe & Central Asia | Sustainability/CSR/MSI |
| Science Based Targets initiative | 2015 | SBTi | London | UK | High income | Europe & Central Asia | Sustainability/CSR/MSI |
| Serving Europe | 1995 | SE | Brussels | Belgium | High income | Europe & Central Asia | Food manufacturing and retail |
| Slovak Association for Branded Products | 1996 | SZZV | Bratislava | Slovak Republic | High income | Europe & Central Asia | Branding and advertising |
| Slovenian Advertising Chamber | 1994 | SOZ | Ljubljana | Slovenia | High income | Europe & Central Asia | Branding and advertising |
| SME Climate Hub | 2020 | SME CH | Stockholm | Sweden | High income | Europe & Central Asia | Sustainability/CSR/MSI |
| SNAC International | 1937 | SNAC Intl. | Virginia | USA | High income | North America | Food manufacturing and retail |
| Sociedad de Fomento Fabril | 1883 | SOFOFA | Santiago | Chile | High income | Latin America & Caribbean | General business and trade |
| Society of European Affairs Professionals | 1997 | SEAP | Brussels | Belgium | High income | Europe & Central Asia | Lobbying, legal and public relations |
| Specialised Nutrition Europe | 1963 | SNE | Brussels | Belgium | High income | Europe & Central Asia | Specialized nutrition and baby food |
| Sustainable Agriculture Initiative Platform | 2002 | SAI Platform | Geneva | Switzerland | High income | Europe & Central Asia | Sustainability/CSR/MSI |
| Sustainable Brands | 2006 | SustBrds | San Francisco | USA | High income | North America | Sustainability/CSR/MSI |
| Sustainable Food Lab | 2004 | SustFLab | Vermont | USA | High income | North America | Sustainability/CSR/MSI |
| Sustainable Food Policy Alliance | 2018 | SFPA | Washington, D.C. | USA | High income | North America | Sustainability/CSR/MSI |
| Sustainable Markets Initiative’s Agribusiness Task Force | 2020 | SMI ATF | London | UK | High income | Europe & Central Asia | Sustainability/CSR/MSI |
| Sveriges Annonsorer | 1924 | SA | Stockholm | Sweden | High income | Europe & Central Asia | Branding and advertising |
| Swiss Association of Nutrition Industries | 2018 | SANI | Bern | Switzerland | High income | Europe & Central Asia | Specialized nutrition and baby food |
| Swiss Food & Nutrition Valley | 2020 | SFNV | Lausanne | Switzerland | High income | Europe & Central Asia | Sustainability/CSR/MSI |
| The American Chamber of Commerce in Vietnam | 1994 | AmCham Vietnam | Ho Chi Minh City | Vietnam | Lower middle income | East Asia & Pacific | General business and trade |
| The Center for Food Integrity | 2007 | CFI | Missouri | USA | High income | North America | Research and science communication |
| The Coordinator of the Food Products Industries - COPAL Argentina | 1975 | COPAL | Buenos Aires | Argentina | Upper middle income | Latin America & Caribbean | General food industry |
| The European Business Network for CSR | 1995 | CSR Europe | Brussels | Belgium | High income | Europe & Central Asia | Sustainability/CSR/MSI |
| The Global Fund | 2002 | TGF | Geneva | Switzerland | High income | Europe & Central Asia | Other |
| The Group DC | 2011 | theGROUP | Washington, D.C. | USA | High income | North America | Lobbying, legal and public relations |
| The Indian Society of Advertisers | 1952 | ISA | Mumbai | India | Lower middle income | South Asia | Branding and advertising |
| The Sustainability Consortium | 2009 | TSC | Arizona | USA | High income | North America | Sustainability/CSR/MSI |
| The Whole Grains Council | 2003 | TWGC | Boston | USA | High income | North America | Primary production, processing and ingredients |
| Thought For Food | 2011 | TFF | Basel | Switzerland | High income | Europe & Central Asia | Sustainability/CSR/MSI |
| TiFN | 1997 | TiFN | Wageningen | The Netherlands | High income | Europe & Central Asia | Sustainability/CSR/MSI |
| Transatlantic Policy Network | 1992 | TPN | Brussels | Belgium | High income | Europe & Central Asia | Lobbying, legal and public relations |
| Tropical Forest Alliance (WEF) | 2012 | TFA WEF | Geneva | Switzerland | High income | Europe & Central Asia | Sustainability/CSR/MSI |
| Tufts University Food and Nutrition Innovation Institute | 2019 | TU FNII | Boston | USA | High income | North America | Research and science communication |
| U.S. Council for International Business | 1945 | USCIB | New York | USA | High income | North America | General business and trade |
| U.S. Global Leadership Campaign | 1995 | USGLC | Washington, D.C. | USA | High income | North America | General business and trade |
| U.S. Plastics Pact | 2020 | USPP | New Hampshire | USA | High income | North America | Sustainability/CSR/MSI |
| UN Food Systems Summit | 2019 | UNFSS | New York | USA | High income | North America | Sustainability/CSR/MSI |
| UNESDA Soft Drinks Europe | 1958 | UNESDA | Brussels | Belgium | High income | Europe & Central Asia | Food manufacturing and retail |
| Union des marques | 1916 | UDM | Paris | France | High income | Europe & Central Asia | Branding and advertising |
| Unione Italiana Food | 2017 | Unione ITL Food | Rome | Italy | High income | Europe & Central Asia | Food manufacturing and retail |
| United Brands Association | 1949 | ABA | Brussels | Belgium | High income | Europe & Central Asia | Branding and advertising |
| United Nations Global Compact | 2000 | UN GC | New York | USA | High income | North America | General business and trade |
| United States Agency for International Development | 1961 | USAID | Washington, D.C. | USA | High income | North America | Other |
| United Way | 1887 | UW | Virginia | USA | High income | North America | Other |
| Wash4Work (UN Glob Compact) | 2016 | W4W | New York | USA | High income | North America | Sustainability/CSR/MSI |
| We Are Still In (WWF, Climate Nexus, and Ceres) | 2017 | We Are Still In | Washington, D.C. | USA | High income | North America | Sustainability/CSR/MSI |
| World Business Council for Sustainable Development | 1995 | WBCSD | Geneva | Switzerland | High income | Europe & Central Asia | Sustainability/CSR/MSI |
| World Economic Forum | 1971 | WEF | Geneva | Switzerland | High income | Europe & Central Asia | General business and trade |
| World Environment Center | 1974 | WEC | Washington, D.C. | USA | High income | North America | Sustainability/CSR/MSI |
| World Federation of Advertisers | 1953 | WFA | Brussels | Belgium | High income | Europe & Central Asia | Branding and advertising |
| World Food Programme | 1961 | WFP | Rome | Italy | High income | Europe & Central Asia | Other |
| World Wildlife Fund | 1961 | WWF | Washington, D.C. | USA | High income | North America | Other |
| World Food Prize Foundation | 1986 | WFPF | Iowa | USA | High income | North America | Research and science communication |
| ZN Consulting | 2003 | ZN | Brussels | Belgium | High income | Europe & Central Asia | Branding and advertising |
